# A Novel Single Pulsed Electromagnetic Field Stimulates Osteogenesis of Bone Marrow Mesenchymal Stem Cells and Bone Repair

**DOI:** 10.1371/journal.pone.0091581

**Published:** 2014-03-14

**Authors:** Yin-Chih Fu, Chih-Chun Lin, Je-Ken Chang, Chung-Hwan Chen, I-Chun Tai, Gwo-Jaw Wang, Mei-Ling Ho

**Affiliations:** 1 Orthopaedic Research Center, College of Medicine, Kaohsiung Medical University Hospital, Kaohsiung Medical University, Kaohsiung, Taiwan; 2 Graduate Institute of Medicine, College of Medicine, Kaohsiung Medical University Hospital, Kaohsiung Medical University, Kaohsiung, Taiwan; 3 Department of Orthopaedics, College of Medicine, Kaohsiung Medical University Hospital, Kaohsiung Medical University, Kaohsiung, Taiwan; 4 Department of Orthopaedics, Kaohsiung Medical University Hospital, Kaohsiung Medical University, Kaohsiung, Taiwan; 5 Department of Orthopaedics, Kaohsiung Municipal Hsiao-Kang Hospital, Kaohsiung Medical University, Kaohsiung, Taiwan; 6 Department of Physiology, College of Medicine, Kaohsiung Medical University Hospital, Kaohsiung Medical University, Kaohsiung, Taiwan; Faculté de médecine de Nantes, France

## Abstract

Pulsed electromagnetic field (PEMF) has been successfully applied to accelerate fracture repair since 1979. Recent studies suggest that PEMF might be used as a nonoperative treatment for the early stages of osteonecrosis. However, PEMF treatment requires a minimum of ten hours per day for the duration of the treatment. In this study, we modified the protocol of the single-pulsed electromagnetic field (SPEMF) that only requires a 3-minute daily treatment. In the *in vitro* study, cell proliferation and osteogenic differentiation was evaluated in the hBMSCs. In the *in vivo* study, new bone formation and revascularization were evaluated in the necrotic bone graft. Results from the *in vitro* study showed no significant cytotoxic effects on the hBMSCs after 5 days of SPEMF treatment (1 Tesla, 30 pulses per day). hBMSC proliferation was enhanced in the SPEMF-treated groups after 2 and 4 days of treatment. The osteogenic differentiation of hBMSCs was significantly increased in the SPEMF-treated groups after 3–7 days of treatment. Mineralization also increased after 10, 15, 20, and 25 days of treatment in SPEMF-treated groups compared to the control group. The 7-day short-course treatment achieved similar effects on proliferation and osteogenesis as the 25-day treatment. Results from the *in vivo* study also demonstrated that both the 7-day and 25-day treatments of SPEMF increased callus formation around the necrotic bone and also increased new vessel formation and osteocyte numbers in the grafted necrotic bone at the 2^nd^ and 4^th^ weeks after surgery. In conclusion, the newly developed SPEMF accelerates osteogenic differentiation of cultured hBMSCs and enhances bone repair, neo-vascularization, and cell growth in necrotic bone in mice. The potential clinical advantage of the SPEMF is the short daily application and the shorter treatment course. We suggest that SPEMF may be used to treat fractures and the early stages of osteonecrosis.

## Introduction

The clinical application of PEMF for treatment of fracture healing has been known for nearly 30 years [1]. Many studies have confirmed the osteogenic effects of PEMF on long bone nonunion repair [1]–[4]. Nevertheless, there are still problems with clinical applications. The main drawback of PEMF treatment is time consumption. The U.S. Food and Drug Administration (FDA) suggested that the stimulating duration of PEMF (EBI Bone Healing System) requires a minimum of ten hours per day for the duration of the treatment. This study aimed to search for a better module of electromagnetic field (EMF) that can more efficiently stimulate osteogenesis for bone repair.

Osteonecrosis (ON) of the femoral head most commonly occurs in young adults aged approximately 20 to 40 years [5]. Without early intervention, the femoral head may collapse, deform, and eventually develop into premature degenerative arthritis. PEMF has been proposed as a nonoperative treatment method for early stage ON [6], [7]. A clinical study from Massari et al. suggested that long-term treatment with PEMF might recover ischemic bone tissue through bone formation and neovascularization in the necrotic area [6]; however, this study still lacks detailed pathological evidence. In the current study, we aimed to investigate whether the newly developed single-pulsed electromagnetic field (SPEMF) can stimulate osteogenesis of bone marrow mesenchymal stem cells (BMSCs) and enhance new formation of bone and vessels, thus preventing ON in its early stages.

We hypothesized that the SPEMF treatment possesses nonhazardous and time-saving properties and may be applied as a treatment for fracture healing and early stage ON without invasive intervention. Based on our hypothesis, we sought an applicable module of SPEMF to enhance osteogenesis and tested the safety of the SPEMF by evaluating the treatment’s cytotoxicity in hBMSCs. We used a noncytotoxic module of SPEMF to test for the potential of osteogenesis, including proliferation and/or differentiation of hBMSCs. In the *in vivo* study, we confirmed the *in vitro* finding by testing the SPEMF effect on bone repair in a necrotic bone graft model in BALB/c mice [8]. Bone repair and neovascularization were evaluated in the grafts bone.

## Materials and Methods

### Ethics Statement

#### Human

The study was approved by the Institutional Review Board (IRB) at Kaohsiung Medical University in Taiwan, and informed consent was obtained from each donor. All participants provide their written consent to participate in this study.

#### Animal

All procedures were approved and performed in accordance with the specifications in the Guidelines of Institutional Animal Care and Use Committee (IACUC) of Kaohsiung Medical University (Permit Number: 100054).

The SPEMF is composed of a single repeated pulse. The pulse’s frequency and magnetic field are adjustable. The pulse’s period is 5 milliseconds (ms) measured in sine waves per stimulation. Each pulse produces the magnetic field, and the magnitude of the magnetic field is adjustable from 0.6 Tesla up to 1 Tesla. Each pulse needs 5 seconds to restore energy for the next pulse. In the first experiment, we tested four different treatment conditions: (1) 0.6 Tesla, 10 pulses per day; (2) 0.6 Tesla, 30 pulses per day; (3) 1 Tesla, 10 pulses per day; and (4) 1 Tesla, 30 pulses per day. The daily treatment was less than 3 minutes. Each treatment condition was tested for 5 days to determine whether SPEMF leads to cytotoxicity in hBMSCs using a lactic dehydrogenase (LDH) assay. If these SPEMF treatment conditions did not cause cytotoxic effect in hBMSCs, then the highest intensity SPEMF was chosen for proliferation and differentiation tests.

### Cell Culture

Bone marrow derived mesenchymal stem cells were obtained from the iliac crest of two different human subjects (one male and one female) as our previous study [9]. Aspirated bone marrow was then layered on a Percoll (Amersham) gradient and centrifuged at 1560 *rpm* for 60 min. The cells in the upper phase were recuperated, centrifuged at 2000 *rpm* for 5 min, and seeded in 15 cm dish with K-NAC medium (Invitrogen-Gibco) at 5% fetal bovine serum (Nalgene), 50 units/ml of penicillin, 50 µg/ml of streptomycin (Invitrogen-Gibco), 0.2 mM of L-ascorbic acid-2phosphate (Sigma), and 2 mM of N-acetyl-L-Cysteine (Sigma) as a selective medium that allows stem cells to keep their self-renewing character [10]. The isolated hBMSCs were amplified and cultured at 37°C with an atmosphere of 5% CO_2_.

### Cytotoxicity Test - LDH Assay

To evaluate whether SPEMF would cause cytotoxicity in hBMSCs, four different conditions of SPEMF were tested: (1) 0.6 Tesla, 10 pulses per day; (2) 0.6 Tesla, 30 pulses per day; (3) 1 Tesla, 10 pulses per day; and (4) 1 Tesla, 30 pulses per day. We repeated 3 times from the same donor (cells from passage number 4 to 7 were used). After 5 days of treatment, the K-NAC medium and cell lysate were analyzed for LDH activity using a cytotoxicity detection kit (Roche). Briefly, each cell culture supernatant and the cell lysate were added to a fresh assay mixture, and the absorbance at 490 nm was recorded. The values were expressed as the sample mean absorbance and normalized to the percentage of the control (without stimulation) value.

### Proliferation Assay - Thymidine Incorporation

Cells were plated at a concentration of 40 cells/mm^2^ in 96-well plates. Twenty-four hours after plating (day 0), cells were treated with SPEMF (1 Tesla, 30 pulses per day) in a K–NAC medium for 2 or 4 days. The effect of SPEMF on the proliferation of hBMSCs was detected at day 2 and day 4. A 2 mCi/well of [^3^H] thymidine was added to each well and incubated for 4 hours prior to harvest. Incubations were terminated by washing with a phosphate-buffered solution (PBS). Cells were detached using 1% trypsin/EDTA and collected in a 96-well UniFilter (Packard, Meriden) using a FilterMate Harvester (Packard, Meriden). The UniFilter was dried with 95% ethanol for 30 minutes. After sealing with TopSeal-A (Packard, Meriden, CT), liquid scintillant was added into the sealed UniFilter and counted in a Top Count Microplate Scintillation and Luminescence Counter (Packard, Meriden, CT). The results were normalized to the percentage of the control value.

### Osteogenic Differentiation of hBMSCs

To induce hBMSCs into osteogenesis, cells were cultured in a bone medium at the preconfluence stage and then in an osteoinduction medium at the postconfluence stage.

The bone medium contained Dulbecco’s Modified Eagle Medium (DMEM) (Invitrogen-Gibco) with 10% fetal bovine serum (Nalgene), 10 mM nonessential amino acid (Invitrogen-Gibco), 0.01% Vit-C (Invitrogen-Gibco), 50 units/ml penicillin and 50 µg/ml streptomycin (Invitrogen-Gibco).

The osteoinduction medium was a stronger induction medium to induce osteodifferentiation in hBMSCs. The osteoinduction medium was a bone medium with 10 mM beta-glycerol phosphate (Sigma), 0.2 mM L-ascorbic acid-2phosphate (Sigma) and 100 nM Dexamethasone [10].

For experiments to test the effect of SPEMF on osteogenic differentiation of hBMSCs, cells were cultured in an osteoinduction medium. Alkaline phosphatase (ALP) activity and mineralization (Alizarin Red S stain) was examined to represent osteogenic differentiation of hBMSCs. The ALP activity of specific hBMSCs reflects that those osteogenic cells were undergoing terminal differentiation. Mineralization was determined using an Alizarin Red S stain.

To investigate the effect of SPEMF on osteogenesis of hBMSCs, the stimulation of SPEMF were divided into two groups. In the first group, the SPEMF 1–25 group was treated with SPEMF from the preconfluence stage (day 1) to the postconfluence stage (day 25). In the second group, the SPEMF 1–7 group was treated from the preconfluence stage (day 1 to day 7).

The hBMSCs were seeded at a density of 40 cells/mm^2^ per 48-well plate. After plating (day 0) for 24 hours, SPEMF stimulation was initiated. Cells in the preconfluence stage were cultured with bone medium. An osteoinduction medium was used after confluence for mineralization. The data were collected on days 10, 15, 20 and 25.

### Alkaline Phosphatase (ALP) Activity Assay and Total Protein Assay

The hBMSCs were plated 100 cells/mm^2^ in 48-well plates and cultured in bone medium at the preconfluence stage. After confluence, the cells were cultured in an osteoinduction medium and treated with SPEMF. The cells were harvested on Days 3, 5, and 7 after stimulation by rinsing them twice with PBS, adding a lysis buffer containing 0.2% (v/v) of Triton X-100, and detaching them from a plate by using a scraper. Cell lysate was assayed for ALP activity using a chemiluminescent method (Tropix, Applied Biosystems, Bedford, MA, USA). The total amount of protein was determined using a Bio-Rad protein assay kit. The specific activity of ALP was expressed as light unit/mg protein.

### Mineralization Assay –Alizarin Red S Stain

To examine the mineralization, cells were washed twice with distilled water and fixed in 10% formalin for 15 min. Cells were then rinsed twice with deionized water and stained with Alizarin Red S (AR-S) for 10 min. at room temperature. AR-S was prepared in deionized water and adjusted to pH 4.2. After staining, the excessive dye was washed gently with deionized water. Calcification deposits were typically stained red. The deposit was then extracted using a 10% acetic acid/20% methanol solution for 45 minutes at room temperature. Spectrophotometric measurements of the extracted solution were detected at 450 nm.

### Animal Experiments

Balb/C mice were anesthetized with an intraperitoneal injection (3.2 mg/30 g body weight) of Ketamine (Ketalar, Parke-Davis, New Zealand) in combination with (3.7 mg/30 g body weight) of Thiazine-hydrochloride (Rompun, Bayer HealthCare, Germany). A 2-mm length of the middle shaft of the tibia on the right side of each mouse was cut out with a saw. This cut bone was frozen with liquid nitrogen for 5 minutes to mimic an avascular segment of necrotic bone graft. Next, the fragment was placed back into its original site in the tibia and intramedullary fixed with 26 gauge needle, as in our previous study [8]. The wound was closed with 4-0 silk sutures. The mice were divided into three groups: (1) a control group (containing the necrotic bone grafts without any SPEMF treatment), (2) a 1–25 days SPEMF group (1 Tesla, 30 pulses per day from post-operation day 1 to day 25), and (3) a 1–7 days SPEMF group (1 Tesla, 30 pulses per day from post-operation day 1 to day 7). Twelve mice in each experiment group were utilized independently and divided into two observation periods during the2^nd^ and 4^th^weeks after surgery (6 mice in each observation period). In SPEMF groups, the mice received SPEMF stimulation externally on coil plate without anesthesia.

### Soft X-ray Observation

After operation, the operated tibia bone was radiographically examined with soft X-rays (SOFTEX, Model M-100, Japan) at 43 KVP and 2 mA for 1.5 seconds to check the fixation position. In the 2^nd^ and 4^th^ weeks after operation, operated tibia bone was examined again after sacrificed. Appropriate magnification was applied throughout the observation, and the results of the micrographs were compared among all groups together with the control.

### Histological Analysis of Bone Tissue

Hematoxylin-eosin (H&E) and immunohistochemical (IHC) quantitative analysis was employed to check for microchanges of the bone tissue. Prior to H&E and IHC staining, all samples of bone tissue were decalcified [0.5 M EDTA-2H_2_O in DDW (186.1 g/L)] and fixed with 4% paraformaldehyde. These samples were embedded in paraffin wax, and serial 5-µm sections were prepared.

### H&E Staining

Sections were routinely stained with H&E. Under lower power magnification, we defined the counted callus area. The counted callus area was within a 1-mm distance proximal and distal to the bone graft ends. The callus area around the graft bone was measured and the percentage of the bone matrix within the callus was calculated by Image-Pro Plus 5.0 software (Media Cybernetics Inc.MD, USA) and compared with the control group. Under high power magnification, the necrotic bone area was measured, and the number of stained lacunae with cells encapsulated within the necrotic bone were calculated and compared among all samples and the control.

### IHC Staining

IHC staining for the Von Willebrand factor (vWF) was performed as follows. Sections were treated for 9 min with 0.15 mg/L of trypsin in a phosphate buffer with a pH of 7.8 and then incubated overnight at 4°C with a 1∶300 dilution of polyclonal rabbit antihuman vWF antibody (CHEMICON International, Inc.). Goat antirabbit biotinylated immunoglobulin (DakoCytomation, Denmark) was used at 1∶300 dilution as the secondary antibody for 60 min at 37°C. An avidin-biotin-peroxidase complex (Vector Laboratories, Burlingame, CA was applied at 1∶300 dilutions for 60 min at 37°C. Peroxidase activity was detected using 0.4 mg/L of 3, 3′-diaminobenzidine in phosphate buffer at a pH of 7.3 in the presence of 0.12 percent H_2_O_2_. Then, the sections were counterstained with hematoxylin. Under high power magnification, the necrotic bone area was measured, and the number of stained endothelium vessels within the necrotic bone were calculated and compared among all samples and the control.

### Statistics

*In vitro*, every experiment of each donor was repeated in triplicate, and data (expressed as mean ± SD) derived from each donor are shown. *In vivo*, 3 sections of histological staining in each mouse were examined and averaged calculated. Analyzer was blinded to the group during quantification. Statistical significance was evaluated by one-way analysis of variance (ANOVA), and multiple comparisons were performed by Scheffe’s method. A p<0.05 was considered significant.

## Results

### Cytotoxicity Assay

**Figure 1:**
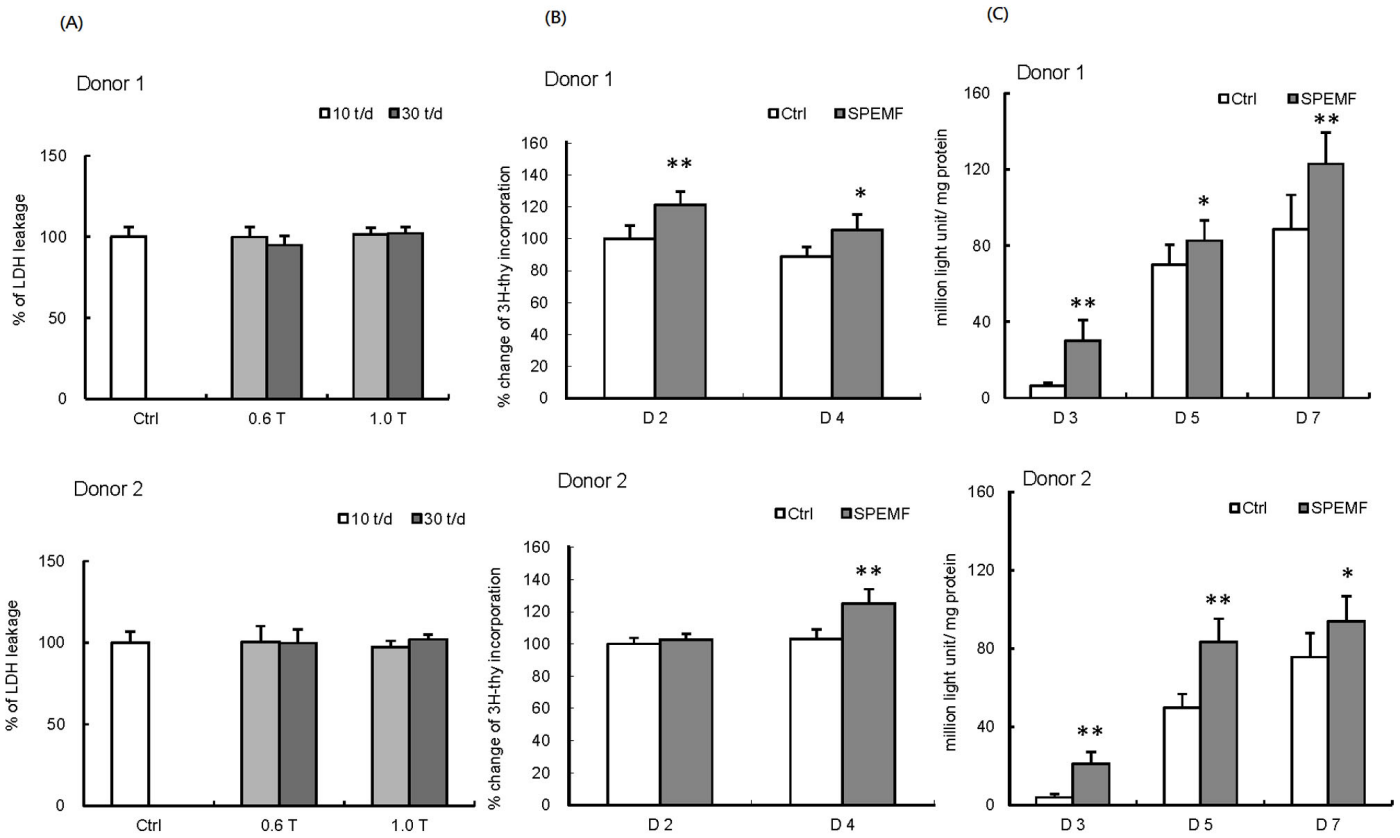
Confirmation of SPEMF stimulating parameter, and SPEMF effects on proliferation and ALP activity. (A) SPEMF has no significant cytotoxic effect on hBMSCs. SPEMF stimulated hBMSCs, both with 0.6 Tesla, 10 or 30 pulses per day and 1 Tesla, 10 or 30 pulses per day, did not lead to cytotoxicity after 5 days of treatment. (B) SPEMF increases proliferation of hBMSCs. The proliferation of hBMSCs was increased after 2 to 4 days of SPEMF treatment: donor 1 shows increase at day 2 and 4; donor 2 shows increase on day 4. (C) SPEMF increases ALP activity of hBMSCs cultured at day 3, 5, and 7. (* p<0.05; ** p<0.01 compared with control).

The data showed that using four modules (0.6 Tesla, 10 pulses per day; 0.6 Tesla, 30 pulses per day; 1 Tesla, 10 pulses per day and 1 Tesla, 30 pulses per day) of SPEMF stimulation for five days did not cause significant cytotoxic effect on hBMSCs from both donors (Fig. 1, A). Therefore, the highest intensity (1 Tesla magnetic field and 30 pulses per day) was used for the following studies.

### Proliferation Assay

After a 2-day SPEMF stimulation, hBMSCs from donor1 increased thymidine incorporation (p<0.01). However, those from donor2 showed no significant change in comparison with the non-stimulated control group (Fig. 1, B). After a 4-day stimulation, the proliferation in hBMSCs from both donors significantly increased (donor 1, p<0.05; donor 2, p<0.01).

### Alkaline Phosphatase (ALP) Activity Assay

hBMSCs cultured in osteo-induction medium showed significantly increased ALP activity by SPEMF stimulation in both donors at day 3 (donor 1, p<0.01; donor 2, p<0.01), day 5 (donor 1, p<0.01; donor 2, p<0.05) and day 7 (donor 1, p<0.01; donor 2, p<0.05) compared to the control group (Fig. 1, C).

### Mineralization Assay

**Figure 2:**
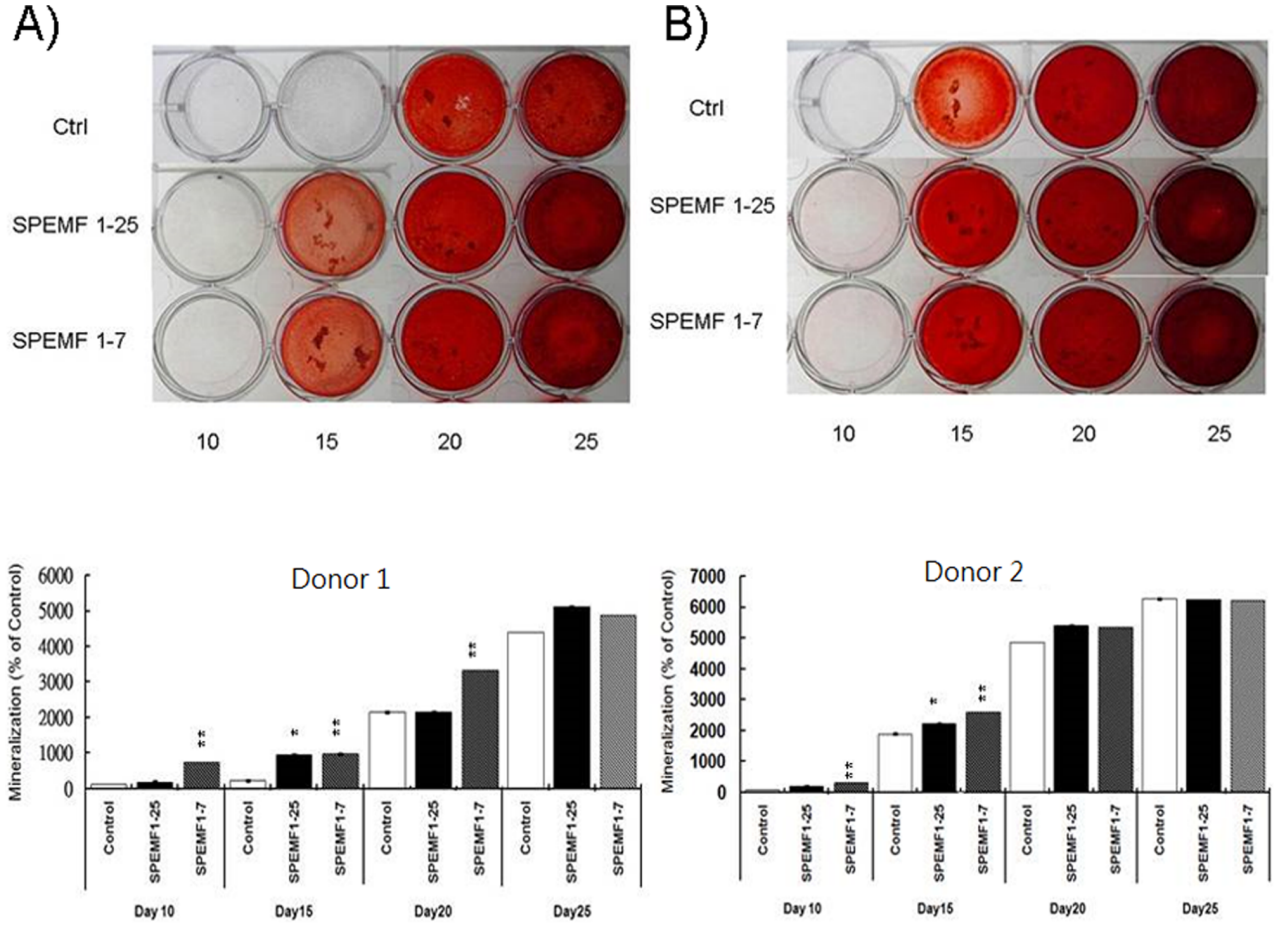
SPEMF increases the mineralization of hBMSCs. SPEMF 1–25 groups were stimulated with SPEMF from day 1 to day 25, while SPEMF 1–7 groups were stimulated from day 1 to day 7. Calcification deposits were collected on the 10^th^, 15^th^, 20^th^, and 25^th^ day. (A) The mineral deposit of donor 1 hBMSCs was increased by SPEMF 1–7 stimulation on the 10^th^, 15^th^ and 20^th^ days, while by SPEMF 1–25 stimulation on the 15^th^ day. (B) The mineral deposit of donor 2 hBMSCs was increased by SPEMF 1–7 stimulation on the 10^th^, and 15^th^ days, while by SPEMF 1–25 stimulation on the 15^th^ day. (P<0.05 *; P<0.01 **).

Calcification deposits were collected on day 10, 15, 20 and 25. Data showed that mineralization of hBMSCs was significantly increased in both the SPEMF 1–7and SPEMF 1–25 groups over these 2 donors at day 15, and both groups showed similar effects (all p<0.01) (Fig. 2). All of the control and SPEMF-treated groups were significantly mineralized at day 20 and 25. No significant difference among these three groups was found.

### Soft X-ray Results

**Figure 3:**
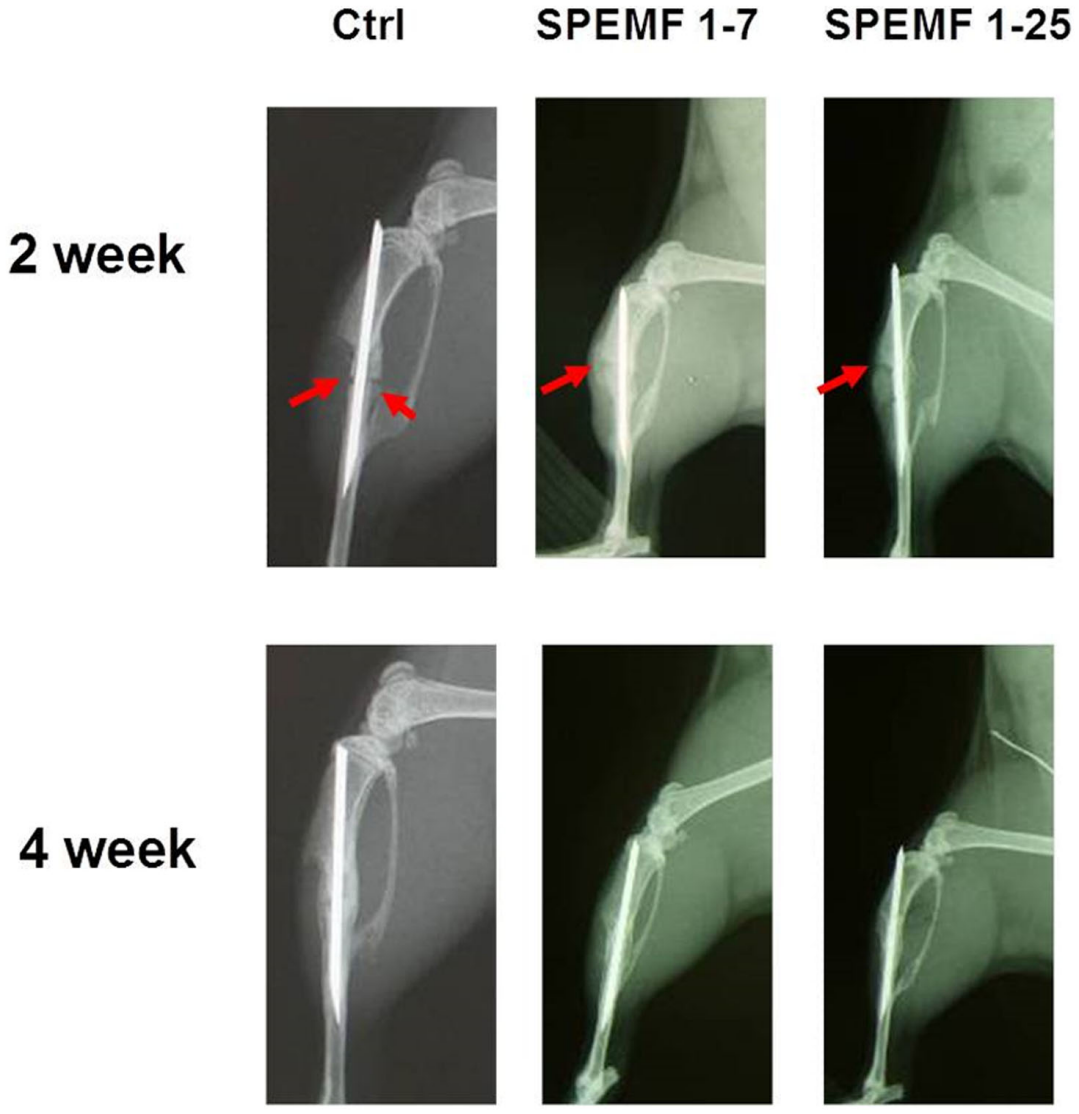
Radiography image of necrotic bone at 2 and 4 weeks after SPEMF stimulation. Both SPEMF groups show increased bridging callus formations over the posterior aspect of the tibia and only a slight radiolucent gap over the anterior tibia portion (red arrows) at the second week. Bone union at the fourth week was complete for all three groups.

Fig. 3 shows the x-ray photographs of the tibia bone graft fragment that was sacrificed at the 2^nd^ and 4^th^ week after surgery. In the control group, the bone ends of the graft were sharp and without obvious callus formation after 2 weeks of surgery. Both SPEMF-treated groups showed better treatment effect than the control. At the 2^nd^ week, we could observe bridging callus formations over the posterior aspect of the tibia and only a slight radiolucent gap over the anterior tibia portion (red arrows). At the 4^th^week, all of the bone ends united completely, and we did not observe any difference between the SPEMF 1–7 and SPEMF 1–25 groups.

### H&E Staining

**Figure 4:**
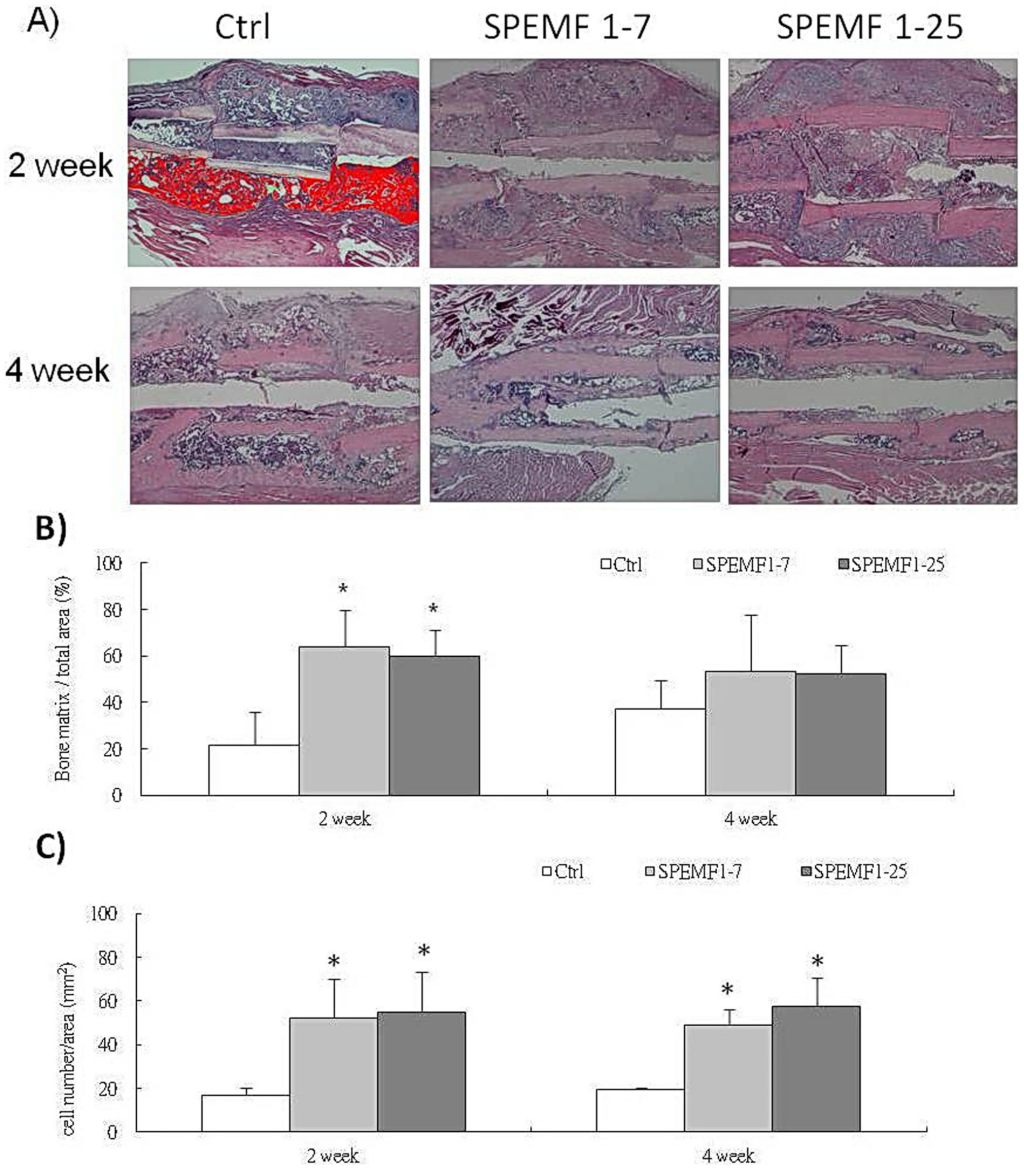
H&E stain image 2 and 4 weeks after SPEMF stimulation. (A) Bone matrix/total area was quantified by Image-Pro Plus 5.0 software (Media Cybernetics Inc.MD, USA). The counting field is 1 mm distance from proximal and distal fracture end. The green line area is callus area. Red colored area in upper left figure indicates the upper half of bone matrix. (B)The result reveals that osteoid increased both in the SPEMF 1–7 group and SPEMF 1–25 group at 2 weeks. (P<0.05 *; P<0.01 **). (C) Cell numbers/area (mm^2^) increased after SPEMF stimulation at 2 weeks and 4 weeks within the grafted bone. (* p<0.05; ** p<0.01 compared with control).

As shown in Fig. 4A, the graft bones, were observed at the 2^nd^ and 4^th^ week. From the micrographs, the SPEMF groups appeared to have had better bridging callus around the graft bone. Four weeks after surgery, calluses bridged the fracture area around the grafted bones. The total callus area amount had no difference in all groups. However, the percentages of bone matrix in both SPEMF-treated groups were significantly elevated at 2^nd^ week compared to the control as shown in Fig. 4 B. In Fig. 4 C shows that the percentage of lacunae with cells encapsulated in both of the SPEMF-treated groups is significantly higher than the control.

### IHC Staining

**Figure 5:**
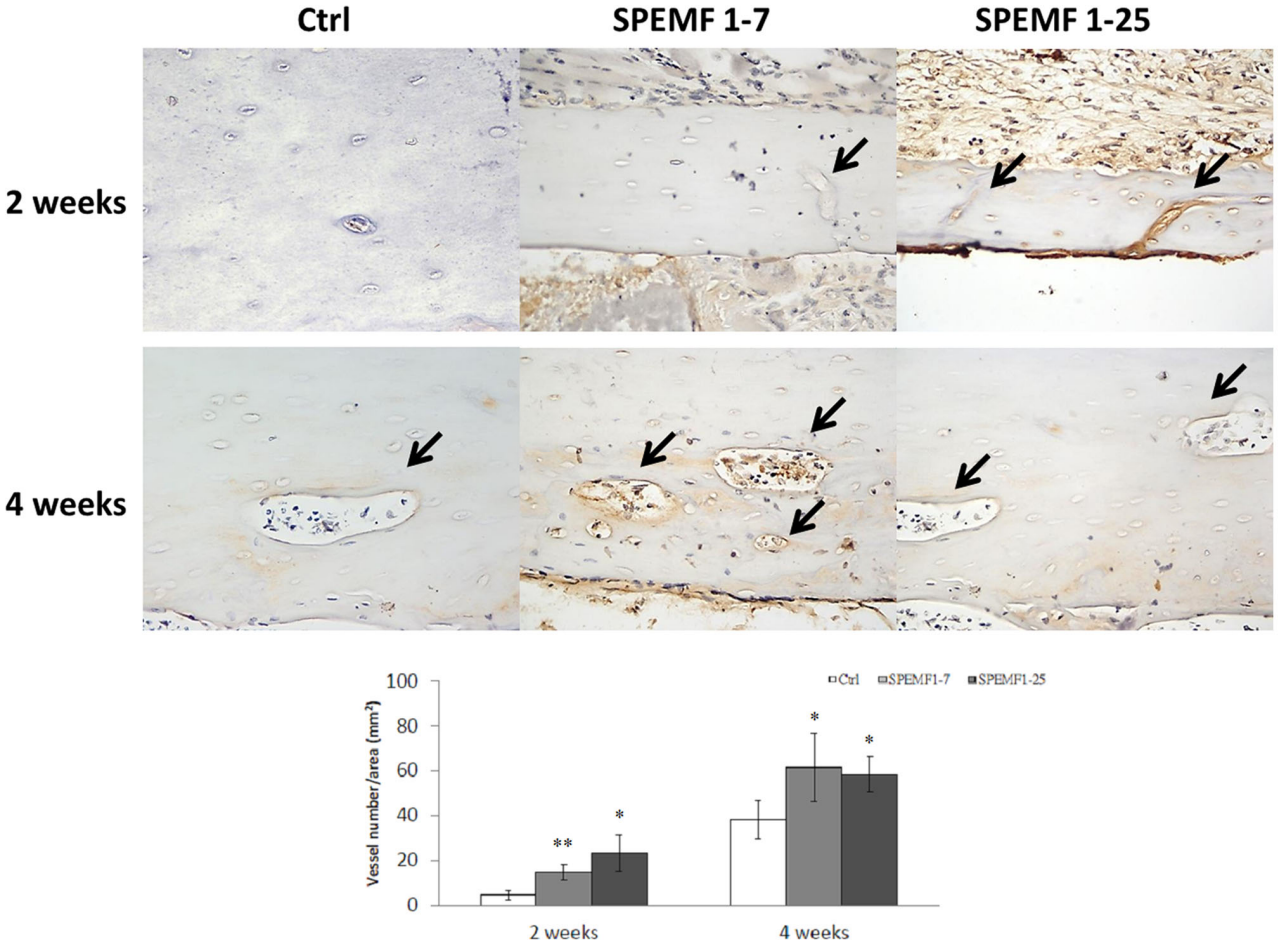
The image of vWF immunohistochemical staining of necrotic bone at 2 and 4 weeks after SPEMF stimulation. (**A**) Black arrows indicate the small vessels inside of graft bone (with 400x magnification). (B) Vessels numbers/area of graft bone increased after SPEMF stimulation at 2 weeks and 4 weeks. (P<0.05 *; P<0.01 **).

After the SPEMF treatment, the formation and growth of new blood vessels are displayed in brown by vWF staining (with high power magnification). Fig. 5A shows that both SPEMF-treated groups had more had more immunoreactivity for vWF in the grafted bone segment. Additionally, large amounts of invading vessels were noted in the grafted necrotic bone. However, only a minimal amount of brown-colored new vessels lined the surface of the graft bone for the control group after 4 weeks treatment. The Fig. 5B shows that the percentage of new vessels with cells encapsulated in both of the SPEMF-treated groups is significantly higher than the control group.

## Discussion

Since 1979, the U.S.FDA has approved the Electro-Biology International Medical Systems (EBI) Bone Healing System for the treatment of nonunion fractures, failed arthrodesis, osteoarthritis [11], osteoporosis [12], ON of the femoral head [13] and spinal fusion [14]. Although clinical applications were successful, some *in vitro* studies on BMSCs found that PEMF enhanced proliferation, but not differentiation, during the exponential phase [15]. However, another study indicated that extremely low-frequency PEMF stimulation induced osteogenesis at early stages of hBMSC differentiation, but suppressed proliferation of hBMSCs [16]. Other than the effect of PEMF on bone fracture repair, Bassett et al. reported that PEMF limited the progression of ON in the femoral head [13]. Massari et al. also suggested that PEMF treatment may be applied at the early stages (stage I and II) of ON in the femoral head [6]. From these previous studies, the biological mechanism of EMF on MSC remains unclear, and the critical timing and time period for EMF intervention on bone repair and/or ON requires further investigation. Our newly developed SPEMF not only stimulates cell growth at the proliferation stage but also enhances osteogenesis at the differentiation stage in cultured hBMSCs. Furthermore, we also demonstrated that SPEMF improves bone callus formation, neovascularization and cell in-growth in the bone graft with a strategy of 3 minute per day for 7 days in a mouse bone graft model.

The primary concern regarding the application of a physical treatment is biological safety. Some reports indicated that higher intensity and frequency of EMF may cause harmful effects to humans; however, these effects have not been proven [17]. Previous reports showed that static magnetic field (SMF) stimulation (1 to 10 Tesla, 0.5 hour to 4 days) did not cause functional damage or cycle progression [18]–[20]. Another report stated that exposure to static magnetic fields alone has no harmful effects on cell growth or genetic toxicity, regardless of the magnetic density [21]. Clinically, magnetic resonance imaging (MRI) is a standard medical imaging tool that uses an intensity of 0.1 to 3 Tesla [22] with no harmful effects to patients [23], [24]. In current study, our *in vitro* study proved that SPEMF (1 Tesla, 30 pulses with a single pulse per day for 7 days) treatment enhanced osteogenesis and had no cytotoxic effect in the hBMSCs.

Another concern of physical stimulus is uncontrolled cell proliferation, which may cause carcinogenesis. Previous studies of PEMF showed discrepant effects on proliferation *in vitro* using different modules of PEMF on different cell lines [25]–[28]. PEMF stimulation at 15 Hz, 18 G was reported to increase proliferation of osteoblast-like cells (MG-63) [25], but not osteocyte-like cells (MLO-Y4) [26]. Another report indicated that PEMF stimulation at 15 Hz, 13 G decreased the proliferation of osteosarcoma cells (SaOS-2) [27]. In current study, the SPEMF (1Tesla, 30 times with a single pulse) increased both proliferation (2–4 days treatment) and osteogenic differentiation (7–15 days treatment) in hBMSCs. Although the SPEMF increases the proliferation of hBMSCs, it also stimulates their ALP activity and mineralization, indicating that the SPEMF enhances osteogenesis of hBMSCs without uncontrolled mitosis. More importantly, we found that the stimulatory effect of SPEMF treatment for 7 days on osteogenesis revealed similar effects to SPEMF treatment for 10, 15, 20 and 25 days. This result indicates that a 7-day, short-course SPEMF treatment optimally enhanced osteogenesis in BMSCs. The SPEMF treatment for 25 days did not have a cytotoxic effect, increasing the possibility of a safe clinical application.

Previous *in vivo* studies of PEMF effects on osteogenesis yielded no conclusive results for clinical application. Taylor et al. suggested that PEMF enhances the healing of complicated fractures to increase vascularity, rather than to directly enhance osteogenesis [29]. Eyres et al. indicated that PEMF has no effect on bone formation, but does prevent bone loss adjacent to the distraction gap [30]. Our *in vivo* study showed that the callus formation significantly increased in comparison to the control 2 weeks after the SPEMF treatment. From this finding, we suggest that the SPEMF treatment may be used at early stages of bone regeneration to enhance callus formation, providing earlier stabilization and better bone unions for bone grafts.

In current study, neovascularization of an osteonecrotic bone was studied using IHC staining to detect vascular endothelial cells. The grafted necrotic bone was pretreated with liquid nitrogen and acted as an osteoconductive scaffold. In the non-SPEMF treated control group, a small amount of vessels was found on the surface of the necrotic bone 4 weeks after grafting. This result indicates that the physiological healing process began at the surface. In the SPEMF-treated groups, not only were there many regenerated vessels scattered on the necrotic bone surface, but large amounts of vessels invading the necrotic bone were also noted. From this result, we demonstrated that SPEMF stimulated neovascularization in the necrotic grafted bone at the early stages of transplantation. This circulation improvement may lead to BMSC recruitment and nutrient supplement. Our histological study further showed that the osteocytes grown in lacunae within necrotic bone in bothSPEMF groups were significantly more than those in the control group. This finding indicated that SPEMF treatment might lead to faster regeneration of dead bone. Accordingly, our *in vivo* study suggests that SPEMF stimulates callus formation and neovascularization earlier than without treatment. In turn, stimulation may facilitate earlier stabilization of the graft and the creeping substitution process.

In conclusion, our results showed that a short-term (3 minutes/day for 7 days) SPEMF treatment enhanced bone healing and increased neovascularization and cell ingrowth within necrotic bone. We propose that SPEMF, the noninvasive physical therapy, may be used to enhance fracture healing and early stage ON with short daily applications and a short treatment course clinically. In the future, we will investigate the influence of SPEMF on bone mineral density of normal mice and the possible negative effects on healthy bone tissue.
